# Intracerebral Hemorrhage Secondary to Solitary Fibrous Tumor: A Case Report

**DOI:** 10.7759/cureus.102549

**Published:** 2026-01-29

**Authors:** Ramón Castruita Meza, Mijail O Quintero Romero, Mauricio D Arteaga Parra, Christian Félix Montiel, Jorge A Cantu Hernandez, Luis F Manzano Romero, Jesus A Morales Gómez, Angel R Martínez Ponce de León

**Affiliations:** 1 Neurosurgery and Neurological Endovascular Therapy Service, Hospital Universitario Dr. José Eleuterio Gonzalez, Monterrey, MEX

**Keywords:** hemangiopericytoma, intracerebral hemorrhage, mesenchymal neoplasm, neuro-oncology, solitary fibrous tumor

## Abstract

Solitary fibrous tumors (SFTs) of the central nervous system are uncommon mesenchymal neoplasms that may remain clinically silent until they reach a significant size or produce neurological manifestations. Although these tumors are typically slow-growing, they can occasionally present with acute neurological deterioration when associated with intracerebral hemorrhage. Such presentations are uncommon and can make diagnosis and management particularly challenging.

We report the case of a 46-year-old woman with no previous medical history who experienced sudden-onset severe headache, vomiting, and rapid neurological decline, ultimately requiring emergent airway protection. On arrival, she presented with a markedly depressed level of consciousness, anisocoria, absent pupillary responses, and severe hypertension. Brain computed tomography (CT) revealed a large left fronto-temporo-parietal intracerebral hematoma producing mass effect and midline shift. The patient underwent emergency decompressive craniectomy with evacuation of the hematoma, during which an underlying tumor was identified and completely resected.

Histopathological examination demonstrated a collagen-rich lesion composed of cellular nodules with elevated mitotic activity, without necrosis or marked atypia. Immunohistochemical staining showed strong nuclear STAT6 expression, supporting the diagnosis of SFT.

This case illustrates the potential for SFTs to present with acute intracerebral hemorrhage, leading to abrupt clinical deterioration. The hemorrhagic event likely contributed to the patient's severe neurological status at admission and prompted urgent surgical intervention. Complete resection was feasible and allowed for histological diagnosis.

Given the possibility of delayed recurrence and the unpredictable behavior of these tumors, long-term postoperative surveillance remains essential. This case highlights the importance of considering underlying neoplasms in spontaneous intracerebral hemorrhage and emphasizes the role of timely surgical management in improving diagnostic accuracy and clinical outcomes.

## Introduction

Solitary fibrous tumor (SFT) (formerly known as solitary anaplastic fibrous tumor/hemangiopericytoma) is a rare mesenchymal neoplasm that typically arises from the dura mater and is defined by the NAB2-STAT6 gene fusion, which drives abnormal pericytic cell proliferation [1]. According to the current World Health Organization (WHO) brain tumor classification, these tumors are graded from 1 to 3 based on mitotic activity, cellularity, and necrosis [2], which correlate with biological behavior.

Intracranial SFTs represent less than 1% of primary central nervous system (CNS) tumors and may mimic meningiomas radiologically, making diagnosis challenging [3]. Nuclear STAT6 immunoreactivity is the diagnostic hallmark [1,2]. While many SFTs exhibit a slow-growing and relatively benign course, higher-grade lesions carry a greater risk of local recurrence and distant metastasis [2,4]. Complete surgical resection remains the cornerstone of treatment, and adjuvant radiotherapy is generally considered in cases with aggressive histology or subtotal resection [5,6].

In this report, we present a case of an intracranial SFT with an uncommon and rarely documented clinical presentation. We highlight its clinical, radiologic, and histopathologic features alongside management considerations.

## Case presentation

A 46-year-old woman with no previous medical history was admitted with sudden-onset intense headache, right-sided significant muscular weakness, and three episodes of vomiting. The patient developed acute respiratory distress, needing airway management through orotracheal intubation, and had a Glasgow Coma Scale (GCS) score of 5, presenting a pupil size of 2 mm and 5 mm in the right and left sides, respectively, with a bilateral absent light reflex, generalized augmented tendon reflexes, and high blood pressure (190/100 mmHg).

**Figure 1:**
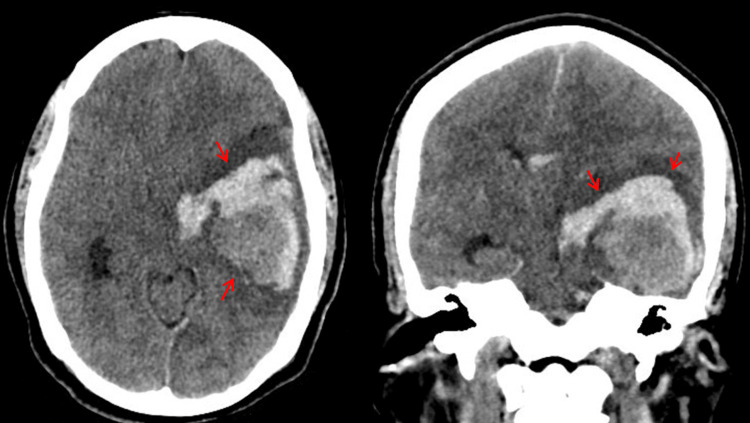
Non-contrast skull computed tomography showing the presence of a left fronto-temporo-parietal parenchymal hematoma Axial and coronal non-contrast brain computed tomography demonstrating a left fronto-temporo-parietal intraparenchymal hematoma measuring approximately 41 cc in volume, with heterogeneous hyperdensity, surrounding hypodense edema, and mild mass effect on the adjacent cortical structures (red arrows).

A non-contrast skull computed tomography (CT) scan revealed an intracerebral hematoma within the left fronto-temporo-parietal region, causing a mass effect and midline shift (Figure 1).

**Figure 2:**
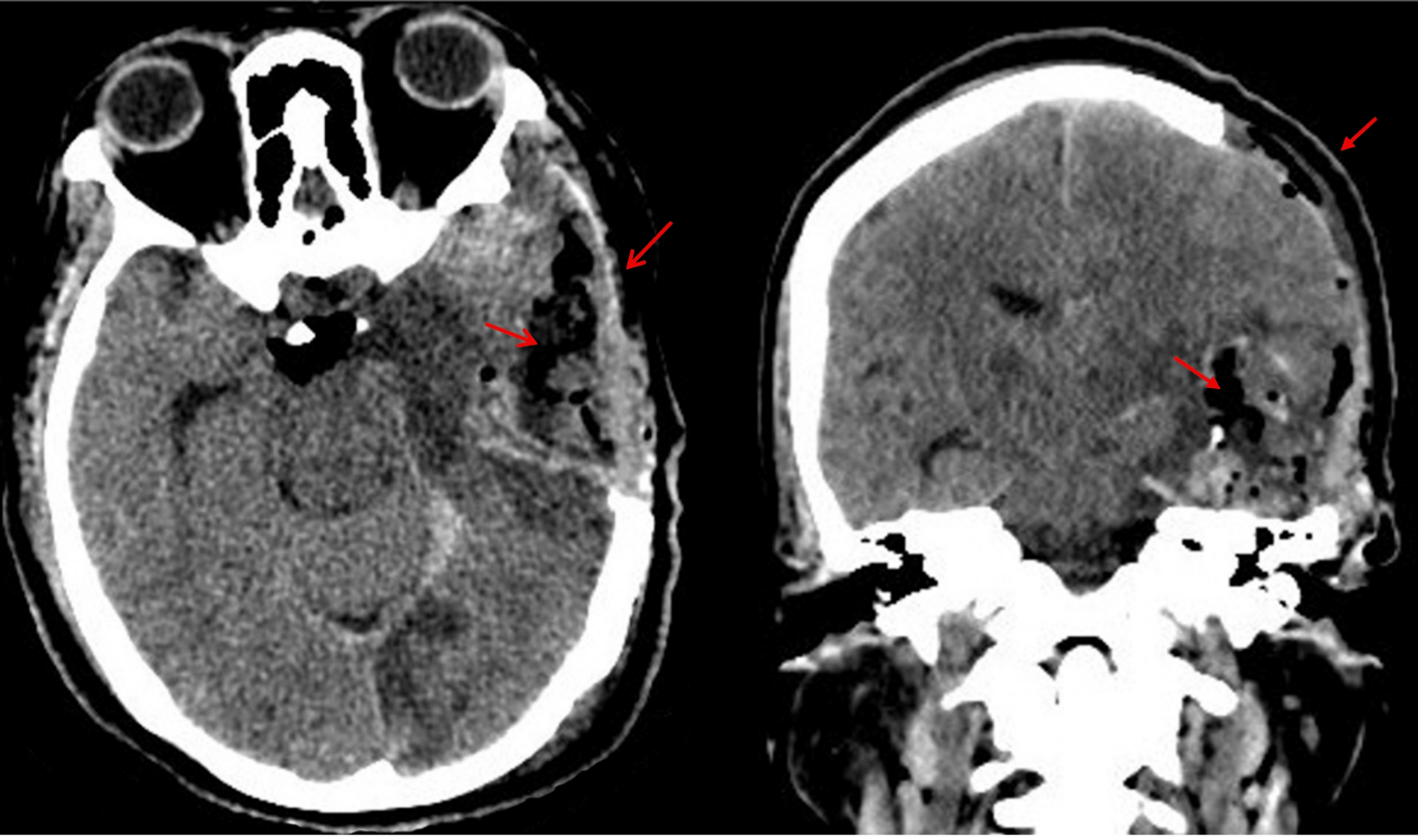
Postoperative non-contrast skull computed tomography scan showing no residual injury Follow-up axial and coronal non-contrast brain computed tomography showing postoperative changes in the left fronto-temporo-parietal region, with a surgical cavity, residual pneumocephalus, resolution of the previously noted midline shift, and no evidence of residual hematoma or acute intracranial hemorrhage (red arrows).

**Figure 3:**
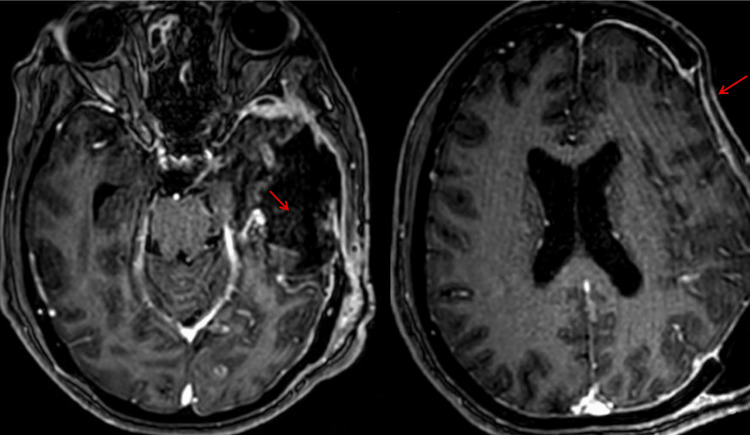
Follow-up brain magnetic resonance imaging (simple and contrast) showing postoperative changes with no residual injury Follow-up axial brain magnetic resonance imaging demonstrating postoperative changes in the left fronto-temporo-parietal region, with a post-surgical cavity and mild linear peripheral enhancement, without evidence of residual lesion, recurrent hemorrhage, abnormal mass enhancement, or midline shift (red arrows).

An urgent decompressive craniectomy was performed, and the hematoma was evacuated. During the procedure, a tumoral mass was observed, which was resected and sent as a pathology sample (Figures 2-3).

**Figure 4:**
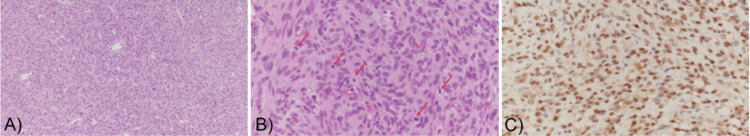
Pathological findings of solitary anaplastic fibrous tumor/hemangiopericytoma (A) Histological sections stained with H&E. Low-power view: The lesion is composed of a neoplasm with a hypocellular to moderately cellular pattern, consisting of a proliferation of spindle-shaped cells arranged in a disorganized pattern, with alternating hypocellular and hypercellular areas. The stroma is fibrous to collagenized, and numerous thin-walled vessels are identified. (B) Histological sections stained with H&E. High-power view: The tumor cells are spindle-shaped, with scant eosinophilic cytoplasm and oval to elongated nuclei, finely granular chromatin, and inconspicuous nucleoli. Scattered mitotic figures are identified (arrows), without marked nuclear atypia. No areas of tumor necrosis or significant pleomorphism are observed. (C) Immunohistochemistry: The neoplastic cells show strong and diffuse nuclear positivity for STAT6, consistent with NAB2-STAT6 gene rearrangement. This finding is highly specific for solitary fibrous tumor. H&E: hematoxylin and eosin

Histopathological analysis of the tumor revealed a dense stromal pattern conformed by numerous hyalinized bands surrounding cellular nodules. No signs of necrosis or anaplasia/advanced dedifferentiation were found. Immunohistochemical staining revealed that cells were positive for STAT6. Histopathological diagnosis was established as an SFT (STAT6+) (Figure 4).

At the three-month follow-up, the patient showed clinical improvement, with a GCS score of 12, pupils at 3 mm, and improved muscle strength in the left side of the body with rigidity and paresthesia in the right side. Myotatic reflexes were increased, as were bilateral Hoffman's and Trömner's reflexes. The patient is scheduled for a follow-up brain magnetic resonance imaging (MRI), and a consultation with oncology is scheduled to assess treatment with radiotherapy.

## Discussion

Intracerebral hemorrhage secondary to SFTs is rare and poses significant diagnostic and treatment challenges. The NAB2-STAT6 gene fusion, which stimulates major cellular proliferation, is associated with their pathogenesis [1]. Although it is rare, hemorrhage within these tumors can cause serious clinical symptoms [2], as in the current case, which required urgent surgical decompression and resection of the mass due to its hemorrhagic presentation.

Although SFTs are usually benign and slow-growing, some cases show increased mitotic activity associated with aggressive behavior and recurrence [3]. Rapid growth, vascular fragility, and abnormal tumor vasculature have been linked to hemorrhagic SFTs [4]. The patient presented with sudden-onset headache and neurological deterioration, consistent with previously reported hemorrhagic cases [5].

Surgical resection continues to be the standard treatment strategy, since it not only decreases the mass effect but also lowers the probability of recurrence [6]. However, adjuvant radiation may be necessary for certain patients to improve long-term outcomes; especially in cases with incomplete resection or high mitotic indexes, entire or subtotal resections are recommended [7]. Adjuvant post-surgical radiation is a procedure that has been associated with higher rates of survival in aggressive SFTs [8]. It is crucial to remember that the various reported cases have different treatment philosophies and there is ongoing discussion over the relevance of radiation therapy [9].

Prognosis is influenced by tumor grade, extent of resection, and presence of hemorrhage [10]. After surgery, some patients have a full recovery, but others suffer from long-lasting neurological impairments or tumor recurrence. Despite treatment, a substantial percentage of cases in our review had poor outcomes because of tumor growth or recurrence. The patient in the present case continued to have right-sided hemiparesis and motor aphasia despite showing no indications of tumor recurrence at follow-up, highlighting the necessity of ongoing neurological surveillance.

## Conclusions

SFTs rarely present with spontaneous intracerebral hemorrhage, yet when they do, the clinical trajectory can be catastrophic. This case is notable due to the acute hemorrhagic presentation requiring emergent decompressive surgery. This emphasizes the significance of early detection, early surgical intervention, and ongoing surveillance. The primary therapeutic approach is surgical resection, and in cases with high proliferative activity, adjuvant radiotherapy is recommended. Heightened awareness of this presentation may shorten the time to diagnosis and intervention, resulting in better patient outcomes in these infrequent but clinically relevant tumors.
